# Exploring social media and admissions decision-making – friends or foes?

**Published:** 2016-10-18

**Authors:** Marcus Law, Maria Mylopoulos, Paula Veinot, Daniel Miller, Mark D. Hanson

**Affiliations:** 1 Department of Family and Community Medicine, Faculty of Medicine, University of Toronto; 2 Department of Pediatrics, Faculty of Medicine, University of Toronto; 3 The Wilson Centre, Toronto, ON; 4 Independent Research Consultant, Toronto, ON; 5 Department of Psychiatry, Faculty of Medicine, University of Toronto

## Abstract

**Background:**

Despite the ever-increasing use of social media (e.g., Facebook, Twitter) little is known about its use in medical school admissions. This qualitative study explores whether and how social media (SM) is used in undergraduate admissions in Canada, and the attitudes of admissions personnel towards such use.

**Methods:**

Phone interviews were conducted with admissions deans and nominated admissions personnel. A qualitative descriptive analysis was performed using iterative coding and comparing, and grouping data into themes.

**Results:**

Personnel from 15 of 17 Canadian medical schools participated. A sizeable proportion had, at some point, examined social media (SM) profiles to acquire information on applicants. Participants did not report using it explicitly to screen all applicants (primary use); however, several did admit to looking at SM to follow up on preliminary indications of misbehaviour (secondary use). Participants articulated concerns, such as validity and equity, about using SM in admissions. Despite no schools having existing policy, participants expressed openness to future use.

**Conclusions:**

While some of the 15 schools had used SM to acquire information on applicants, criteria for formulating judgments were obscure, and participants expressed significant apprehension, based on concerns for fairness and validity. Findings suggest participant ambivalence and ongoing risks associated with “hidden” selection practices.

## Introduction

As social media (i.e., websites and applications, such as Facebook, Twitter, Instagram, blogs, discussion forums) have grown in reach and use, their presence has become felt in a vast array of contexts. Social media provides a variety of platforms for users to express themselves, and to view the expressions of others, with few limitations. Particularly among youth, social media is often used as a popular form of self-expression and socializing. Contemporary youth may view their online and personal identities as inseparable, without, perhaps, reckoning with the full real-world implications of unbridled self-expression.

In medical education, a number of concerns have been raised relating both to social media’s expressive reach and its public accessibility, in particular the professionalism of trainees’ online behaviours.^1^–^4^ These concerns include real and potential violations of patient confidentiality, use of profanity, discriminatory language, depiction of intoxication, and posting of sexually suggestive material.^1^,^2^

To address these concerns, various physician and medical trainee associations have produced guidelines and standards specifying appropriate and professional social media use.^5^–^12^ However, such guidelines and standards for trainees and practitioners do not establish and make transparent school standards and policies regarding social media use, nor do they relinquish the responsibility of faculties of medicine to educate trainees about this relatively new dimension of professionalism.^6^ An example of this is the need to adopt deliberate, ethical, and accountable practice when using digital media.^13^ There is, in fact, evidence that such policies and standards have not been established. Research in the United States indicated that of its 132 accredited medical schools, only 13 had student guidelines that explicitly mentioned social media, and were accessible online.^2^

Despite this absence of transparent, structured policy at the school level, there is nevertheless evidence that social media *is* being used in the selection of applicants in a variety of higher education settings, including those outside of medicine,^3^,^14^–^16^ as well as in medical school and residency admissions.^17^–^19^ For example, Schulman et al.^18^ found, among a sample of 600 US medical school admissions officers and residency program directors that 19% reported using Internet searches to find information on students and 9% used social networking sites to evaluate candidates.

The fact that social media is being used in admissions should not, perhaps, be a surprise, given its public accessibility. It is difficult to imagine how the use of social media could be eliminated in admissions, even if this were deemed desirable. However, there also appears to be a near total absence of established and transparent standards for the use of social media profiles in the admissions process.^18^ Whether social media profiles should be included as a part of candidates’ formal admissions applications, or whether admissions directors should have the right to investigate social media regarding concerning behaviours of candidates that come to light during the application process is unclear. The obscurity of social media’s influence on admissions is problematic, particularly in the absence of official policy, given the potential for selection bias.^18^ In addition to privacy protection, we should be concerned about the transparency of its use in admissions,^20^,^21^ insofar as we believe that medicine and higher education are built on public trust, and the belief that the best and brightest will form the future generation of practitioners.^22^

The evidence base on the use of social media in medical school admissions is limited. Aside from limited evidence on use of social media and availability of policy, Canadian data are sparse, and even less is known about how social media are being used and the attitudes towards use in the medical school admissions process. As a means of expanding this nascent field of inquiry, and to provide a framework for future research, we broadly sought to understand the use and potential value of social media in the medical school admissions process. We hoped to:

1. Determine whether social media presence is being reviewed during the admissions process in Canadian medical schools;
2. Describe *how* and *why* social media presence is being reviewed, insofar as it is;
3. Explore participants’ attitudes regarding the morality and utility (or value) of reviewing social media presence during the admissions process;
4. Determine whether schools have formal policies regarding review of applicants’ social media presence during the admissions process.

## Methods

### Study design and outcome measures

Qualitative descriptive methodology was used for this exploratory work as we were interested in developing rich, but straight, descriptions of the subject matter.^23^,^24^ It is a pragmatic approach, with overtones of other qualitative approaches (e.g., phenomenology, grounded theory), that applies a purposeful sampling strategy.^24^ Qualitative description is a flexible approach that is particularly useful in answering questions of importance for practitioners and policy makers, in part because it avoids extensive theorizing, attempting to stay as close as possible to descriptions of participants’ perspectives, as expressed in their own words; this is sometimes referred to as “low-inference interpretation, or likely to result in easier consensus among researchers.”^23^,^24^ “Qualitative descriptive studies have as their goal a comprehensive summary of events in the everyday terms of those events.” (p. 334)^23^ These studies employ a range of data collection strategies (e.g., minimally-to-moderately structured open-ended interviews with individuals, observation, document review).^24^ Data are summarized using content analysis, resulting in a straight description of the data in everyday language^23^,^24^ rather than interpretation vis-a-vis a highly conceptual, philosophical, or abstract framework or system.^23^

### Study sample and setting

Ethics approval was obtained through the University of Toronto Research Ethics Board. Participants were undergraduate admissions deans, directors, chairs, or designated admissions office staff members from Canadian medical schools, purposively sampled^24^,^25^ because of their knowledge and experience, and the centrality of their roles, in their respective schools and admissions offices. To obtain as broad a picture as possible of the situation in Canadian medical education,^24^ we sought to obtain data from personnel from as many of Canada’s 17 medical schools as possible; this included both English and French schools, and a broad geographic representation. We began sampling by emailing the highest ranking position in the admissions office of each school to arrange an interview. If that individual nominated another person with whom to speak, we then contacted that person. Participants representing 15 of 17 Canadian medical schools were interviewed (*n*=17 participants; two schools had two participants). Ten participants were either Chairs or Assistant/Associate Deans of Admission; three were Directors of Admission; and four were in manager or coordinator roles in admissions offices. All participants were interviewed for their leadership role and experience. Participants, regardless of position, were interviewed to obtain information on behalf of the admissions office they represented. When conducting the interviews we clarified that this was the viewpoint we were seeking (i.e., description of actions conducted on behalf of the admissions office, rather than for personal reasons).

### Data collection

One interview was conducted simultaneously with two individuals; the remainder were all one-on-one. Telephone interviews were conducted by one of the authors (PV), an experienced interviewer with no current or previous relationships with participants. Interviews took place over a three-month period in 2014, using a semi-structured interview guide (Table 1). Interviews were audio-recorded and transcribed verbatim. Transcripts were entered into NVivo 10 (QSR International, Melbourne, Australia) for organization and data analysis. In keeping with qualitative descriptive methodology, we developed an interview guide that was relatively structured and minimally theoretical,^24^ incorporating team members’ practical experiences and expertise in medical school admissions (ML, MH), undergraduate medical education, and technology in education (ML). The guide was iteratively refined, through the addition of probes, as interviews were conducted and analyzed. Expansion and restructuring of the interview guide allowed for organization of the interview to ensure that the interviewer explored the core topics. At the same time, additional impromptu probe questions were employed to allow participants to elaborate on these topics, as required. As the interviews progressed, we reordered and expanded some of the questions. For example, we elaborated our questioning on ethics and fairness. We added questions to obtain a better sense of what led participants to look up information on an applicant on social media in the first place. We also inserted specific questions related to the presence of organizational policy to inform decision-making about selection of candidates for medical school.

### Data analysis

Data analysis involved iterative, line-by-line, coding and grouping of data into relevant categories by one of the authors (PV), with minimal theoretical elaboration,^23^ and in regular consultation with the research team. Continuous review (PV, ML, MM, MH) was used to add rigour, with the team collectively reviewing and discussing the findings and codes. The eventual coding scheme was approved (PV, ML, MM, MH) as adequately representing the relevant data. Analysis occurred iteratively with data collection. As a complement to the predominantly qualitative analysis, responses on three topics were also counted^23^ to obtain a sense of the number of schools in which applicants’ social media presence had been considered: in general, as a means of *selecting* applicants, and as a means of *deselecting* (rejecting) applicants.

Response counting was also conducted to better understand the prevalence of formal policies on the use of social media to inform admissions and whether participants generally thought that social media have a legitimate role to play in admissions.

## Results

Many of the schools we looked at had examined, at some point, social media profiles to acquire information on applicants. Participants reported using Facebook, Twitter, various online video platforms, Google Groups, and other online forums to look up information on medical school applicants. None of the participants reported using it explicitly to screen all applicants (primary use); however, a sizeable proportion did admit to looking at social media to corroborate preliminary indications of worrisome behaviour (secondary use). In the absence of guidance (none of the schools had policies), participants articulated concerns, such as validity and equity, about using social media in admissions. Participants expressed openness to future use and policy implementation (albeit a watchful waiting approach) thus mitigating these issues.

### Using social media for corroboration of concerns

While participants representing several schools reported that applicants’ social media profiles were examined during the admissions process to gain additional information, none reported using social media to deliberately evaluate applicants for the express reason of selecting candidates (primary screening), including for the purpose of identifying positive applicant attributes. However, several participants reported inclusion of information found on social media in criteria used to reject applicants, as a means of following up on worrisome indications.

Some participants thought that social media had potential utility or value for screening out candidates following the identification of concerns during standard admissions processes. In this context, it was viewed as a secondary screening procedure (Participant 05), used only as needed. Several participants suggested that secondary screening, but not primary use, may be feasible and justified. The excerpts below are descriptions of *actual use*, but participant attitudes can be clearly inferred:

If somebody has made a comment in the letter of reference that is worrisome, or a file reviewer has flagged that person for something they found in the application itself, or things that come up at the interviews that people flag as worrisome behaviour, we will go and sometimes look and see if we can find their social media presence to see if there is anything there that corroborates that concern. (Participant 01)

It’s a second-level thing that I think it might be useful at, but we have used it only in one single case in the last five years. And that applicant, just for your information, had posted very strange, racist, and bigoted comments. That was what was coming back from the referee. When we looked it up, the referee was absolutely correct. So, that applicant was dead in the water. (Participant 05)

…the most recent example was a complaint from a number of our existing medical students, about someone they knew who was applying to medical school at our institution. So these were unsolicited complaints that came forward, and the complaints were driven by the fact that these current students had seen things written in social media, posted by this applicant, that they felt were unprofessional. So we intentionally then went and tried to verify that information firsthand, so we then looked up this applicant’s social media site, it was primarily Facebook… Well, it generated an activity that wouldn’t have otherwise have occurred, and the activity was to request a formal response to our concern. (Participant 12)

Conversely, one participant felt that it was precisely this kind of *ad hoc* use that was problematic, and suggested that if social media were incorporated at all in admissions, it needed to be used as a primary screen of all applicants, as described previously:

It is what is currently in vogue, but I would just want to be careful before we would ever go down that road to make sure that whatever we use that it’s done fairly. If it’s done for one person, it has to be done for everybody […] I couldn’t imagine that it would be allowed if it was just done in a haphazard or ad hoc way. (Participant 08)

While it was challenging for participants to pinpoint benefits, potential pitfalls were more readily articulated. Among these was concern for process transparency, informed consent, applicant privacy, and validity or truthfulness (being able to trust the information as factual) as a source of selection criteria. In one example, the participant appeared to indicate overlap between the issues of privacy and valid decision-making, suggesting that, without clear criteria for deciding *how* judgments were to be made, there would be no limit to the extent of prying:

On a systematic basis of looking up every applicant, personally I feel like it’s a bit of a violation of their privacy although it is public [...] Can we make real judgements about people based on these things? How much digging do we need to do before we’re confident that they’re good enough to be a medical doctor? (Participant 08)

### Concerns about the validity of social media

Participants expressed uncertainty about the validity of social media use as a selection tool and questioned the evidence-base for engaging in this practice:

…can you actually rely on what people say about other people or what people say about themselves as being truthful or factual? (Participant 05)

It’s not evidence based, […] can’t be verified or refuted, and the principles of due process wouldn’t be in place. (Participant 07)

Certainly before we adapt or adopt anything new, we do like to have a fairly good research background or grounding for whatever it is we’re going to introduce. […] I would hope that anything that is coming forth for an admissions process is properly researched. (Participant 13)

Participants had concerns for the questionable accuracy of information and the means of drawing inferences from social media. Among these was the possibility of confusing an applicant with someone else of the same name:

There could be multiple people with the same name, and you don’t know what they look like so how do you know it’s the right one that you have? (Participant 09)

Many participants also felt that it is difficult to accurately interpret information when the surrounding circumstances are not readily apparent (i.e., out of context):

…it was interesting because when we actually said to the applicant, we found this and we’re concerned, have you got anything to say, he pointed out to us that it was actually produced as part of a professional acting class that he was taking. (Participant 01)

Somebody could take a 10 or 15 second clip that could look absolutely awful, but if you don’t know the circumstances around it, maybe it was part of a play, but maybe somebody has posted it that makes it look as if it’s something happening in real life. (Participant 07)

Some also noted that applicants are generally young, may not always think through the ramifications of personal postings on social media, and may not appreciate the potential damage resulting from what may be a momentary lapse in judgement:

…often, when we’re dealing with younger individuals, they might not have a good social filter, and when using social media, they may post things that could be viewed as immature or inappropriate. I think that could leave a negative impression if someone from admissions or recruitment was looking them up, that could be an issue. (Participant 10)

I feel that young people today are so caught up with it and they don't think of what they’re putting out there as permanent. It’s obvious that they don’t think about it… (Participant 13)

Some evoked potential legal implications, the issue of liability, and the importance of due diligence:

The only thing is that if you’re going to use information from secondary sources, I think to avoid litigation and lawsuits and various other things you have to make sure it’s validated. (Participant 05)

There’s no doubt, though, that the admissions process needs to be transparent, it needs to be an equitable process, and there can’t be hidden agendas. It’s more and more open to challenges, and sometimes legal challenges, from applicants. So that’s driving a process that is regulated by fairly formal policies. (Participant 12)

### The importance of ensuring an even playing field for applicants

The issue of procedural equity overlapped with the issue of valid decision-making. This overlap was embodied in the concept of bias, in which one applicant may be favoured over another in an unfair manner:

If you want to have a very equitable admissions process, by looking up an applicant you are automatically going to learn more about their gender and maybe their ethnicity and things like that. It could create a bit of a bias […] not everyone has access to social media or not everyone uses it. (Participant 10)

…our philosophy is, whatever tools we use, must be applied in an equitable way across our application pool, and that we’re attempting, at least, to assess the same qualities and attributes amongst the entire application pool. (Participant 12)

I think that if we were to think about doing that we would have to certainly notify applicants that that was being done […] Again, the only moral or ethical issue I would have is if it was being used without the knowledge of an applicant. (Participant 03)

A few participants noted a lack of resources (admissions staff, time, money) to search candidates on a broad scale in order to ensure equity, particularly given unknown benefit:

To be honest with you, we’ve considered doing a routine screen out there. But I need staff to do that, and I don’t have staff to do that. You need time, and time is people, and people is money. (Participant 05)

On a systematic basis of looking up every applicant […] it’s just very hard to operationalize doing something like that on a more broad level... Yes, it would take a lot of time and resources. (Participant 08A)

Many participants were open to considering a role for social media in selection, but with caveats, as described above. Even those who did not perceive, or were unsure of, a potential role for social media in the selection process were not averse to considering its possible use, and accompanying guidance:

I can’t see it right now. I can answer no for 2014, but I certainly have my eyes and ears open to change. (Participant 11)

Some did not think a policy was urgently needed at this time. Most preferred a watchful waiting approach, suggesting that policy would be needed in the future as this issue gained prominence. Indicating both a watchful waiting approach, and recognition of some kind of significance, participants were also interested in what other schools were doing and recognized value in developing official policies:

Yeah, I think if it becomes more prevalent and more institutions are using it so then applicants are curious or wondering if it would be the case with our institution, it’s always nice to have a policy or something there that makes your process transparent. (Participant 06)

## Discussion

This study helps further our understanding of the use of social media in admissions in medical schools. It also has relevance to residency admission practices, given similar issues related to the need for transparency, fairness, and the possibility of “hidden” selection practices. A notable feature of our findings was the tension between the observations of a large number of participants who thought that social media had a legitimate, or at least potential, role in admissions decision-making, and the difficulty they had articulating the value of using it for this goal. This may reflect a similar tension between the facts that, while having looked up applicants’ social media presence was a fairly common practice, the legitimacy for this was questionable, even from the perspectives of participants. Not only did the participating schools not have any official policies regarding this, but there was a degree of obscurity and contradiction in why and how applicants’ social media presence was examined, and when in the process it was used.

The finding that secondary screening (i.e., for corroboration) was the most commonly reported actual use, raises the questions of what specifically leads admissions personnel to examine a potentially troublesome social media presence in the first place, and how this examination is then used in relation to preliminary suspicions. The description by one participant that review of social media is directed towards whether or not there is ‘corroboration,’ for example, raises questions about the extent we have to prove the accuracy.

Even if the social media use is appraised without prejudice, the act of viewing it may produce undue bias because, with such review, a variety of personal information becomes accessible - sexual orientation and political opinions, for example.^4^ Exposure to this information may produce unanticipated and hidden bias that, while officially unsanctioned, could still influence the evaluation of applicants, detracting from the validity and equity of the process, all while hidden from view. This is problematic insofar as it is in the interests of the medical profession, and the public, to make medical school accessible to highly qualified applicants, regardless of background, and to produce a workforce that is capable of meeting a range of needs, employing a variety of perspectives, and working in a diversity of settings.^21^,^22^

There is a reasonable expectation of the right to a certain degree of honest expression and self-representation when using social media. Users, particularly young adults, are unlikely to have anticipated the full range of audiences who can or will view this information,^26^,^27^ much less exert control over expressions made when they were adolescents. It is unfair and unproductive to punish them simply for not having done so - a potential and unfortunate side-effect of the indelibility of online material.

Moreover, the very justification for examining social media for selection purposes depends on the assumption that we can thereby access honest disclosures. This was highlighted by our participants’ concern for lack of context that may lead to erroneous assumptions about applicants’ social media content. Yet, by examining social media presence without clear criteria for how this should and should not be used to inform judgments, we risk selecting specifically for those who appear “squeaky clean” just by avoiding direct and honest expression. Schulman et al.^18^ suggest that guidelines for professional behaviour on social media sites may be of value in assisting applicants to avoid rejection due to bias. Implied in this suggestion is recognition of the potential for the professionalism concept to extend into questionable domains, potentially discouraging free and honest expression and selecting against traits that are irrelevant, or positive, in relation to applicants’ potential as future physicians. For this reason, the watchful waiting approach preferred by many of our participants is concerning.

The fact that some participants reported the use of social media as a means of connecting to applicants in a personal way is significant from the perspective of fairness and validity. Implied in this rationale is an assumption that social media is a valid and accurate representation of true identity. Cain^4^ notes that social networking sites are often understood in precisely this way - as facilitators of self-presentation, articulation of identity, and social interaction. While features, like friends’ lists and privacy controls, allow users to exert some control over content and audience, the persistence, replicability, and searchability of online content make it difficult or impossible to predict the audiences who will, at some point, view one’s expressions, or the manner in which they will interpret them.^26^

The main limitation of this study is the reliance on reports from single (in two cases, two) admissions personnel as measures for general practices in their respective settings. We also did not ask all participants whether they used social media to look up multiple candidates. This may have resulted in an underrepresentation of the extent of use, since individual reports do not tell us whether others involved in the process (i.e., members of the selection committee) may also have reviewed applicants’ social media presence. Similarly, the types of use reported, and attitudes towards use may not be representative of a given school/admissions office. Further research is required to assess the experiences, practices, and attitudes of a more complete range of personnel within institutions.

### Conclusion

The findings of this study indicate that a sizeable proportion of Canadian Medical School admissions offices had, at some point, examined social media profiles to acquire information on applicants. Most participants expressed that using social media as an across-the-board screening tool (primary use) was not desirable, with objections including privacy violation and lack of feasibility. More generally, participants identified a variety of concerns about the use of social media in admissions. The main concerns participants expressed were violation of applicant privacy, equity of the admissions process, and the validity of decision-making. Our findings stand as a cautionary tale. In the absence of any sort of direction on this issue, the risk is that social media will be used to reproduce inequalities; that is, without guidance, hidden practices may inadvertently threaten to exert bias, resulting in lack of transparency and a degradation of fairness in the admissions process.

## Figures and Tables

**Table 1:** Interview guide

	**Have you looked up an applicant to medical school on social media? Please note by social media, we are referring to applications such as Facebook, Instagram, Twitter, and/or any other interactive online media?**	
**If YES Probes**	What drove you to look up the applicant’s social media profile?	What social media applications did you use?
	What information did you find?	Did that information you found on social media influence whether or not you found a candidate suitable for medical school?
	Did the information you found influence your perceptions of the applicant’s professionalism?	Are you aware of other individuals in the selection process (e.g., student file reviewers or interviewers, faculty file reviewers or interviewers) looking up applicants on social media?
**If NO Probes**	What is your opinion on looking up applicants on social media?	Are you aware of others in similar positions who have looked up an applicant to medical school on social media?
	What are the benefits of looking up applicants on social media?	What are the downfalls of looking up applicants on social media?
	**Does your institution have any formal policies about using information obtained from social media to inform decision making about selection of candidates for medical school?**	
**Probes**	Please describe the policies.	Are all individuals involved in the selection process aware of those policies?
	Do you think there is a need for such policies?	Are there systematic methods of incorporating information obtained from such sources, into the decision making-process?
	**Do you have any moral or ethical issues with the use of social media in the selection process for undergraduate medical school?**	
**Probes**	What has influenced your perspective on this?	Has there been an incident that influenced your perspectives on these issues?
	Do you think that using social media would influence the fairness (or perceived fairness) of the selection process?	
	**Have medical school applicants used or referenced social media in their applications?**	
**Probes**	What applications have they used?	Has their use of social media had an influence on your decision-making?
	**It is increasingly common for employers in workplaces to use social media as a means to screen applicants. What do you think of the use of social media in the hiring process?**	
**Probes**	Is this appropriate? Not appropriate?	In what instances is this practice appropriate?
	What are the potential benefits?	What are the potential pitfalls?
	**Do you think social media has a role to play in the admissions process for medical school?**	
**Probes**	If YES, please describe this role.	If NO, please explain why not.
